# Supplementary material for: Flexible decapyrrylcorannulene hosts
Source: Nat Commun. 2019 Jan 30;10:485. doi: 10.1038/s41467-019-08343-6 (PMC6353959; doi:10.1038/s41467-019-08343-6)
Supplement: Supplementary file 1 — Suplementary Information [file 41467_2019_8343_MOESM1_ESM.pdf]

# Supplementary Information

## Flexible decapyrrylcorannulene hosts

Xie et al.

[1] State Key Lab for Physical Chemistry of Solid Surfaces, Collaborative Innovation Center of Chemistry for Energy Materials, Department of Chemistry, College of Chemistry and Chemical Engineering, Xiamen University, Xiamen 361005 (China)
[2]Hefei National Laboratory for Physical Sciences at Microscale, CAS Key Laboratory of Materials for Energy Conversion, Department of Materials Science and Engineering, Synergetic Innovation Center of Quantum Information & Quantum Physics, University of Science and Technology of China, Hefei 230026, China

E-mail: xmuzhangqy@xmu.edu.cn, sfyang@ustc.edu.cn, syxie@xmu.edu.cn

## Supplementary Methods

All the reaction were carried out under the nitrogen atmosphere. NMR spectra were acquired on a Bruker AV 500 Spectrometer at 298 K in the solvents indicated. $^{1}$H NMR (500 MHz) and $^{13}$C NMR (125 MHz) spectra were recorded using a Bruker AV 500 Spectrometer. Chemical shifts were reported at the delta scale in ppm relative to $CHCl_3$ (δ = 7.26 ppm), toluene-$d_8$ (δ = 7.00 ppm) for $^{1}$H NMR and $CDCl_3$ (δ = 77.0 ppm) for $^{13}$C NMR. $^{1}$H and $^{13}$C NMR spectra are provided for all compounds. Ultraviolet-visible-near infrared absorption spectra were recorded using an Agilent UV-Vis-NIR Cary 5000 Spectrometer. Emission spectra were recorded using a Hitachi F-7000 Fluorescence Spectrometer. Mass spectra were recorded using a Bruker Fourier Transform Ion Cyclotron Resonance Mass Spectromter (FT-ICR MS) and a Bruker time of flight mass spectrometer coupled with matrix-assisted laser desorption/ionization source (MALDI-TOF-MS). Silica gel (300-400 mesh) or alkaline aluminum oxide was used for column chromatography. Unless otherwise noted, materials obtained from commercial suppliers were used without further purification.

## Supplementary Note 1

**Synthesis of Decachlorocorannulene (1)**

Decachlorocorannulene (**1**) is prepared by one step of perchlorination reaction as reported[1].

## Supplementary Note 2

**Synthesis of DPC (2a)**

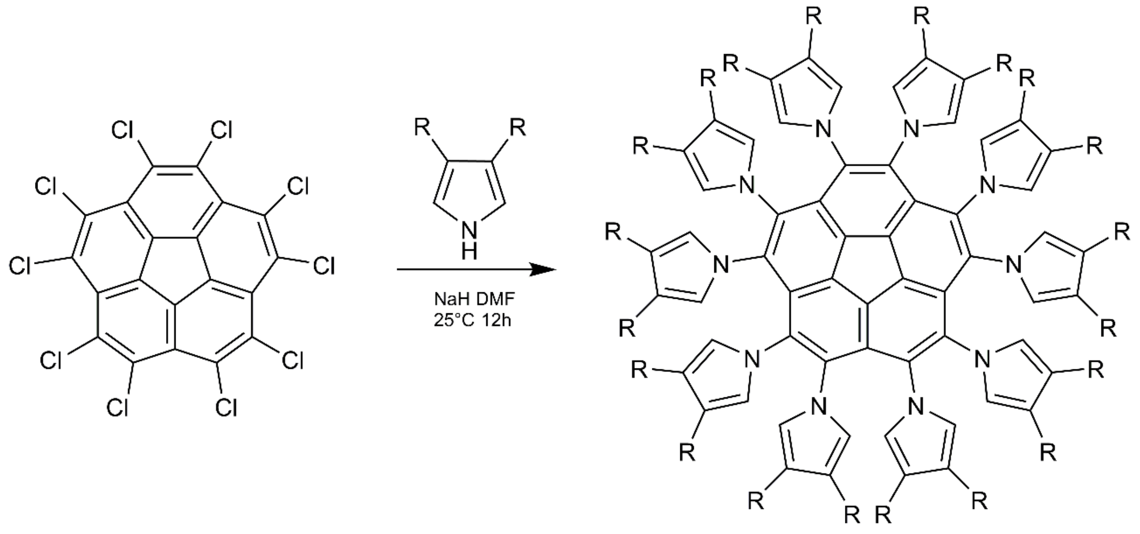


**1** **2**

**2a** R = -H
**2b** R = $-CH_3$

To a DMF (15 ml) solution of NaH (60 % oil dispersion, 80.7 mg, 2.00 mmol), 135.1 mg (2.01 mmol) of

pyrrole was added at 0 ℃. After the evolution of $H_2$ gas has ceased, the reaction mixture was stirred for an additional 30 min at the same temperature, and then decachlorocorannulene **1** (100.0 mg, 0.17 mmol) was added. The reaction mixture was stirred for an additional 12 h at 25 ℃ before it was poured into ice-water (20 ml). Then the mixture was added dichloromethane (20 ml) and the layers were separated, and the aqueous layer was thoroughly extracted with dichloromethane. The combined organic layers were washed with water and brine, and dried over magnesium sulfate. After filtration and evaporation of the solvent, 45.36 mg (0.05 mmol) of **2a** was separated by alkaline aluminum oxide column using $CH_2Cl_2$/hexane (1:1) as the eluent. The isolated yield is approximately 30 %. **2a**: $^1$H NMR (500 MHz): δ = 5.76 (20 H, dd), 6.04 (20 H, dd) ppm. $^{13}$C NMR (125 MHz): δ = 109.86, 122.55, 124.27, 130.41 and 138.45 ppm. HRMS (FT-ICR) *m/z* calcd for $C_{60}H_{41}N_{10}$ $[M+H]^+$: 901.3515, found 901.3521. (See Supplementary Figures 1 and 2).

## Supplementary Note 3

### Synthesis of DPC (2b)

To a DMF (15 ml) solution of NaH (60 % oil dispersion, 136.0 mg, 3.40 mmol), 323.0 mg (3.40 mmol) of 3, 4-dimethylpyrrole was added at 0 ℃. After the evolution of $H_2$ gas had ceased, the reaction mixture was stirred for an additional 30 min at the same temperature, and then decachlorocorannulene **1** (100.0 mg, 0.17 mmol) was added. The reaction mixture was stirred for an additional 12 h at 25 ℃ before it was poured into ice-water (20 ml). Then the mixture was added dichloromethane (20 ml). Then the layers were separated, and the aqueous layer was thoroughly extracted with dichloromethane. The combined organic layers were washed with water and brine, and dried over magnesium sulfate. After filtration and evaporation of the solvent, the products were purified by silica gel column chromatography using $CH_2Cl_2$/hexane (1:1) as the eluent. 40.12 mg (0.034 mmol) of **2b** was collected. The isolated yield is approximately 20 %. **2b**: $^1$H NMR (500 MHz): δ = 2.03 (60 H, s), 6.51 (20 H, d) ppm. $^{13}$C NMR (125 MHz): δ = 30.13, 118.87, 120.12, 129.29, 132.18 and 137.05 ppm. Mass spectrometer coupled with matrix-assisted laser desorption/ionization source (MALDI-TOF-MS): *m/z* = 1180.0 (for $C_{80}H_{60}N_{10}$). Some impurities are shown in the $^1$H and $^{13}$C NMR spectra likely due to instability of **2b** under the ambient conditions, fortunately the single crystal of **2b** was obtained by a fast evaporation approach and the structure of **2b** was further identified by crystallography. (See Supplementary Figures 3 and 4).

## Supplementary Note 4

**Synthesis and HPLC isolation of $C_s$-$C_{71}H_2$-I, $C_s$-$C_{71}H_2$-II, and $C_{2v}$-$C_{71}H_2$-III.**

The $C_{71}H_2$-containing soot was prepared by our homemade setup in acetylene-benzene-oxygen diffusion combustion. The flame was maintained under a pressure of 10-20 torr. The optimized synthetic conditions are given as follows: chamber pressure: 15~20 torr; gas flow rate: $O_2$, 0.55 l/min; $C_2H_2$, 1.10 l/min; hot benzene steam, 1.10 l/min. A large amount of collected $C_{71}H_2$-containing carbon soot were extracted with toluene by ultrasound and filtration. The crude toluene-extracting solutions of the soot are very complex and typically contain lots of PAHs, fullerenes, and fullerene derivatives. Therefore, the desired compound $C_{71}H_2$ isomers were separated by a multiple-stage HPLC process using Cosmosil Buckyprep column and Cosmosil 5PPB column. After several cycles of separation, the final purities of $C_s$-$C_{71}H_2$-I, $C_s$-$C_{71}H_2$-II, $C_{2v}$-$C_{71}H_2$-III are up to 99%. All the separations were performed at room temperature using toluene as mobile phase on a Shimadzu LC-6AD HPLC instrument and the chromatogram was monitored at 330 nm. (See Supplementary Figures 5 and 6).

## Supplementary Note 5

**Detailed descriptions for growth of all co-crystals**

All of these DPC (**2a**)-fullerene co-crystals were grown through solvent evaporation. For $C_{60}$, $PC_{61}P$ or $Sc_3N@C_{80}$, we respectively took a small amount of fullerenes to dissolve in carbon disulfide, and then moved 0.5 ml of the fullerene carbon disulfide solution to a 5.0 ml centrifuge tube, followed by mixing with a certain concentration of dichloromethane solution of DPC to give a mixed solution typically have a mole ratio of DPC and fullerene ~2:1. High-quality co-crystals suitable for X-ray crystal structure analysis can be obtained after keeping the mixed solutions at room temperature for slow evaporation of solvents for several days. For the other fullerenes [$C_{70}$, $C_{90}$, α-$PC_{71}BM$, $\beta_1$-$PC_{71}BM$, $PC_{61}AE$, $C_{60}HPh$, $C_{60}HCH_3$, $C_{71}H_2$-I, $C_{71}H_2$-II, $C_{71}H_2$-III, $C_{65}H_6$, or $(C_{59}N)_2$], we firstly took a small amount of fullerene to dissolve in toluene respectively. Subsequently, a certain concentration of DPC in toluene or dichloromethane was mixed into the toluene solution of fullerene, with the mole ratio of DPC and fullerene ~2:1. Lastly, the mixed solutions were kept at room temperature for several days and high-quality co-crystals suitable for X-ray crystal structure analysis can be obtained after slow evaporation of solvents. Rather than individual solvent toluene or carbon disulfide, the solvent combination such as carbon disulfide/dichloromethane and toluene/dichloromethane is

critical for the co-crystal growth.

It is worth mentioning that all the reported DPC (**2a**)-fullerene co-crystals are stable long enough for X-ray crystallographic identification under ambient air at room temperature. Both the fullerenes and DPC (**2a**) can be recycled through ultrasonic decomplexation for the co-crystals (especially for the carbon disulfide-involving co-crystals), followed by column chromatography or HPLC separation.

## Supplementary Note 6

### Theoretical electronic properties of 2a

To understand the observed electronic properties, theoretical calculations were performed for **2a**. Time dependent (TD) DFT calculation result of **2a** molecule at the B3LYP-D3BJ/6-31G(d) level showing a charge transfer band at λ = 383.79 nm and λ = 383.75 nm are assigned to HOMO-13/ HOMO-14→LUMO/LUMO+1 and HOMO-15/ HOMO-16→LUMO/LUMO+1 transitions (Fig. S8). Interestingly, HOMO-13 and HOMO-14 are double degenerated, the similar cases happen in the HOMO-15 and HOMO-16, LUMO and LUMO+1. The other bands at λ = 270.64 nm and λ = 270.62 nm are assigned to HOMO-10/HOMO-11→LUMO+2/LUMO+3 and HOMO-22/ HOMO-23→LUMO/LUMO+1 transitions. Here, HOMO-10 and HOMO-11, HOMO-22 and HOMO-23 are also double degenerated. (See Supplementary Figures 7-8, Table S1).

## Supplementary Note 7

### $^{1}$HNMR titration study for the supramolecules of DPC (2a) and $C_{60}$ and $C_{70}$. (See Supplementary Figures 12 to 15).

The supramolecular formation of DPC/$C_{60}$ and DPC/$C_{70}$ were investigated by $^{1}$H NMR titration experiment on the basis of the complexation-induced chemical shifts for protons in pyrryl groups. The $^{1}$H NMR titrations were carried out using deuterated toluene as the solvent. The binding constants ($K_a$ or $K_{assoc}$) were estimated from the changes of chemical shift for α-H and β-H applying non-linear curve fitting[2] using the following equation:

$$\Delta\delta = \frac{\Delta\delta_{\max}(1+K_aX+K_aC)-[\Delta\delta_{\max}^2\times(1+K_aX+K_aC)^2-4K_a^2XC\Delta\delta_{\max}^2]^{1/2}}{2K_aC}$$

Where

$X$ = $[C_{60}]_{total}$;

$C$ = $[H]_{total}$, the total concentration of DPC (**2a**);

$\Delta\delta_{max}$ means that $\Delta\delta$ at 100% complexation;

$K_a = K_{assoc} = [H \supset C_{60}] / ([C_{60}] \times [H])$;

$K_{assoc}$ and $\Delta\delta_{max}$ were optimized as parameters in the non-linear curve fitting using Origin© software (v. 9.0).

**Titration of DPC (2a) with $C_{60}$:** A stock solution of DPC (**2a**) was prepared by dissolving 0.278 mg of **2a** in 1 ml of deuterated toluene. 400 µl of $3.00 \times 10^{-4}$ M solution of compound **2a** was subsequently titrated with 20, 40, 60, 80, 100, 200, 400, 600, 800 µl of $2.00 \times 10^{-3}$ M solution of $C_{60}$ in deuterated toluene and the result was presented in Supplementary Figure 12. The estimated $K_a$ values were 9372.57 ± 1497.10 and 7131.04 ± 419.22 $M^{-1}$ for α-H and β-H with $C_{60}$ respectively and the mean $K_a$ is 8251.81 ± 209.66 $M^{-1}$ (see Supplementary Figure 14).

**Titration of DPC (2a) with $C_{70}$:** A stock solution of DPC (**2a**) was prepared by dissolving 0.278 mg of **2a** in 1 ml of deuterated toluene. 400 µl of $3.00 \times 10^{-4}$ M solution of compound **2a** was subsequently titrated with 20, 40, 60, 80, 100, 150, 200, 400, 600, 800 µL of $2.00 \times 10^{-3}$ M solution of $C_{70}$ in deuterated toluene and the result was presented in Supplementary Figure 13. The estimated $K_a$ values were 6151.69 ± 197.87 and 5220.54 ± 40.83 $M^{-1}$ for α-H and β-H with $C_{70}$ respectively and the mean $K_a$ is 5686.16 ± 119.35$M^{-1}$ (see Supplementary Figure 14).

## Supplementary Note 8

### DFT calculations

All the geometry optimizations were calculated at B3LYP-D3BJ[3]/6-31G(d) level except that the LANL2DZ basis set was applied for Sc atoms in $Sc_3N@C_{80}$ system. Based on the optimized geometries, intermolecular interaction energies and inversion barrier energies were obtained by the same density functional theory (DFT) function with 6-31G(d, p) basis set. In both inter- and intra-molecular interaction energy calculation, the basis set superposition error (BSSE) was taken into consideration with the counterpoise (CP) method[4]. All calculations were performed with the quantum chemistry program Gaussian 09 package[5]at 298.15 K, 1 atm and gas-phase. (See Supplementary Figure 17 to 19, Supplementary Table 11 to 14, and Supplementary Data 1).

An intramolecular interaction calculation method[6] was employed for the interactions between two adjacent pyrryl groups of **2a**. The calculation results (Supplementary Table 11) show that pyrryl groups repel each other in both AAP and BAP (Supplementary Figure 18), and the repulsion energy in APP is about 2 kcal/mol larger than that in BAP, such the difference accords well with the geometrical configuration that d(AAP) is shorter than d(BAP) and the two pyrryl groups in BAP are more parallel than that in AAP inferring from the dihedral angle β. The depth of **2a** (0.61 Å) is shallower than corannulene molecule (0.86 Å), which is attributed to the repulsions among the pyrryl groups. Such repulsions can be quantified by $\Delta E^{int}_{AAP}$ and $\Delta E^{int}_{BAP}$ as shown in Supplementary Table 11.

Supplementary Table 13 shows interaction energies (kcal/mol) of representative six co-crystals. Two conclusions can be drawn: 1) The binding energy between (+)DPC or (-)DPC and fullerenes ($\Delta E^{int}_{F/(+)DPC}$ and $\Delta E^{int}_{F/(-)DPC}$) differs mainly due to the interaction between external groups and hosts. 2) Weak intermolecular interactions ($\Delta E^{int}_{(+)DPC/(-)DPC2}$) exist between the V-shaped hosts, but the interaction disappears between the parallel hosts ($Sc_3N@C_{80}$ and $(C_{59}N)_2$ systems).

Theoretical interaction energy between one molecular **2b** and one molecular $C_{60}$ (-34.98 kcal/mol) is slightly larger than that of between **2a** and $C_{60}$ (-31.39 kcal/mol), as reflected in the Supplementary Data 1. In the three molecular packing of **2a**, the cavity suitable for fullerenes entering can be theoretically optimized, complying with the experimental observation in the crystal packing of **2a**. In the three molecular packing of **2b**, by contrast, the key cavity is found to disappear. On the other hand, the interactions of **2b** itself are much larger than that of **2b** and $C_{60}$, as a result, **2b** is inclined to self-assemble rather than assemble with fullerenes according to theoretical prediction, which might be another possible reason for the failure of co-crystal between **2b** and fullerenes. (See Supplementary Figure 19, Supplementary Data 1).

## Supplementary Figures

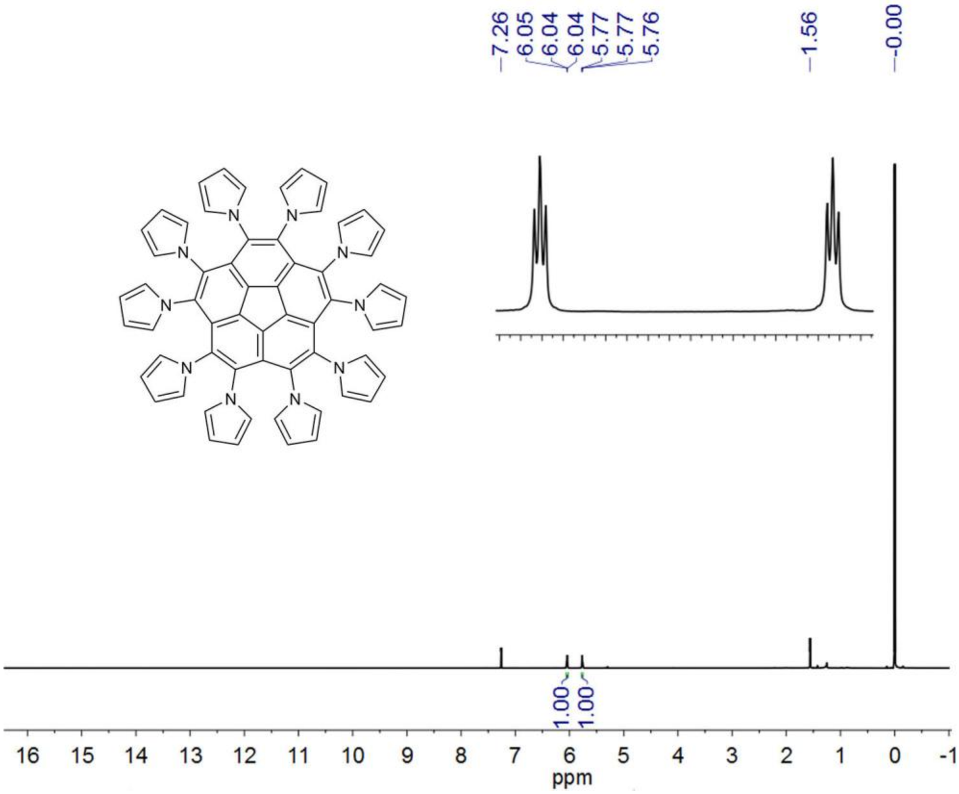


**Supplementary Figure 1.** $^{1}H$ NMR (500 MHz, $CDCl_3$) spectrum of DPC (**2a**).

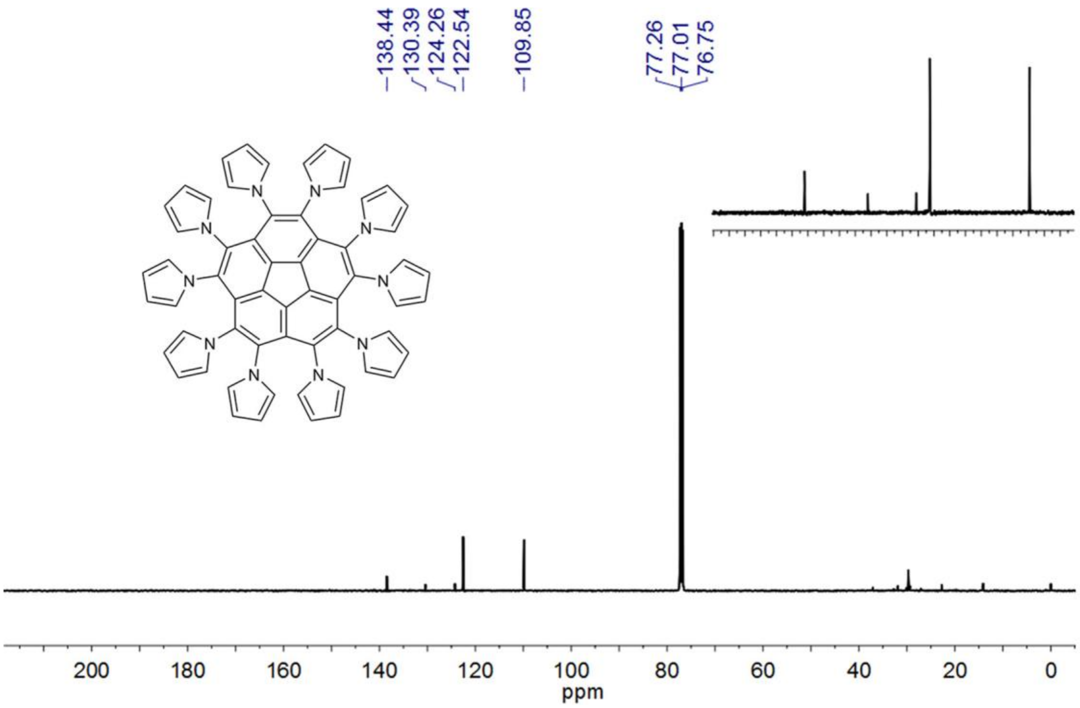


**Supplementary Figure 2.** $^{13}C$ NMR (125 MHz, $CDCl_3$) spectrum of DPC (**2a**).

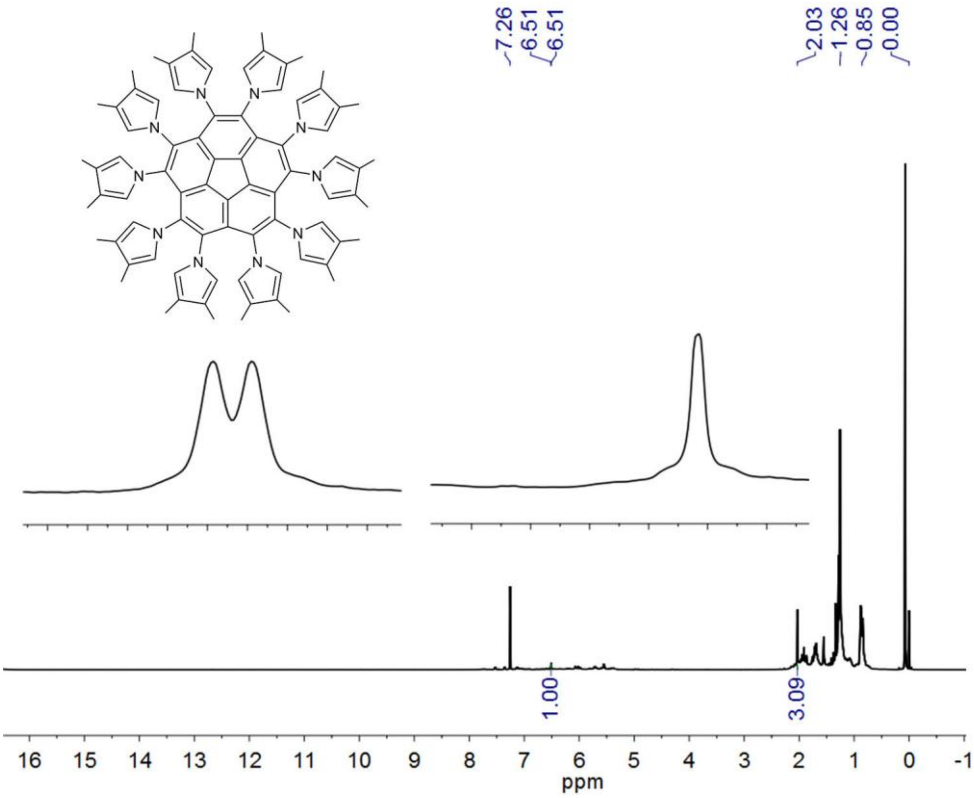


**Supplementary Figure 3.** $^{1}H$ NMR (500 MHz, $CDCl_3$) spectrum of **2b**.

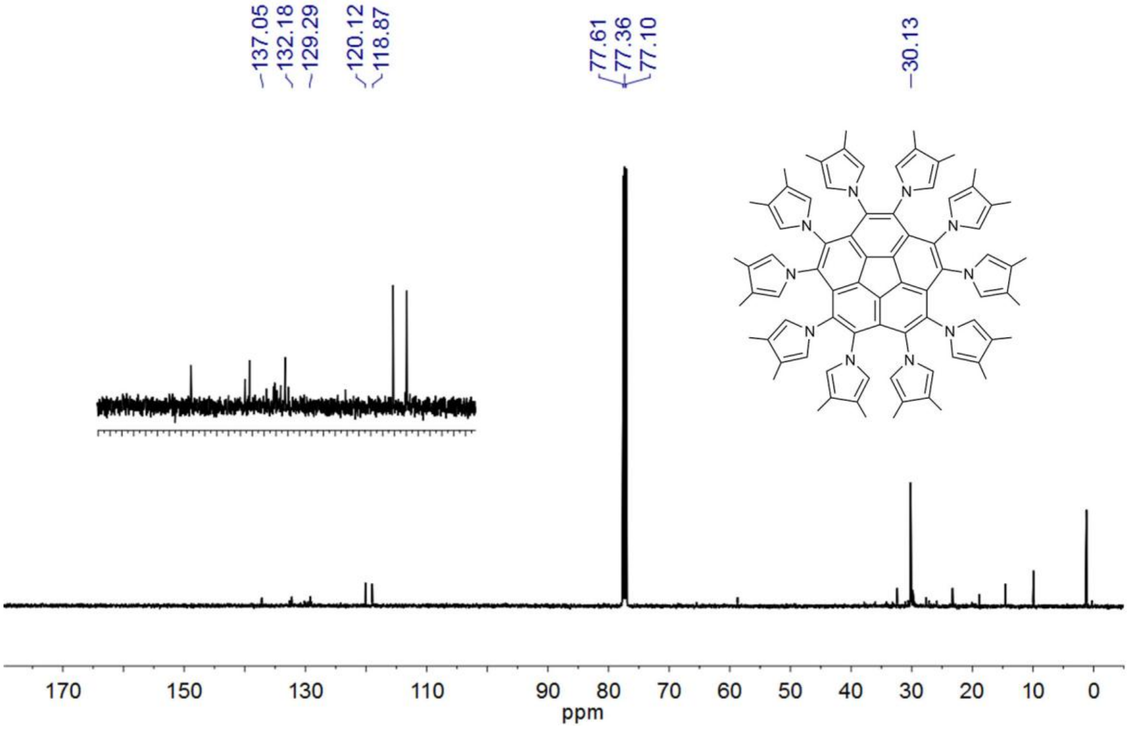


**Supplementary Figure 4.** $^{13}C$ NMR (125 MHz, $CDCl_3$) spectrum of **2b**.

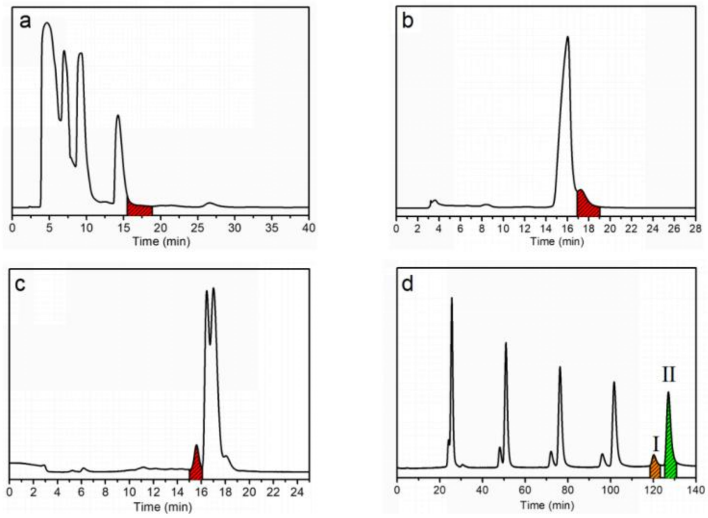


**Supplementary Figure 5. HPLC separation of *$C_s$*-$C_{71}H_2$-I, *$C_s$*-$C_{71}H_2$-II. a**) Column: buckyprep column (20 mm×250 mm), flow rate: 20 ml $min^{-1}$, eluent: toluene. **b**) Column: 5PBB column (10 mm×250 mm), flow rate: 6 ml $min^{-1}$, eluent: toluene. **c**) Column: buckyprep column (10 mm×250 mm), flow rate: 4 ml $min^{-1}$, eluent: toluene. **d**) Column: 5PBB column (10 mm×250 mm), flow rate: 4 ml $min^{-1}$, eluent: toluene. The regions of collected $C_{71}H_2$-containing components are highlighted as red shadow. The region of pure *$C_s$*-$C_{71}H_2$-I is highlighted as orange shadow, and the pure *$C_s$*-$C_{71}H_2$-II is highlighted as green shadow.

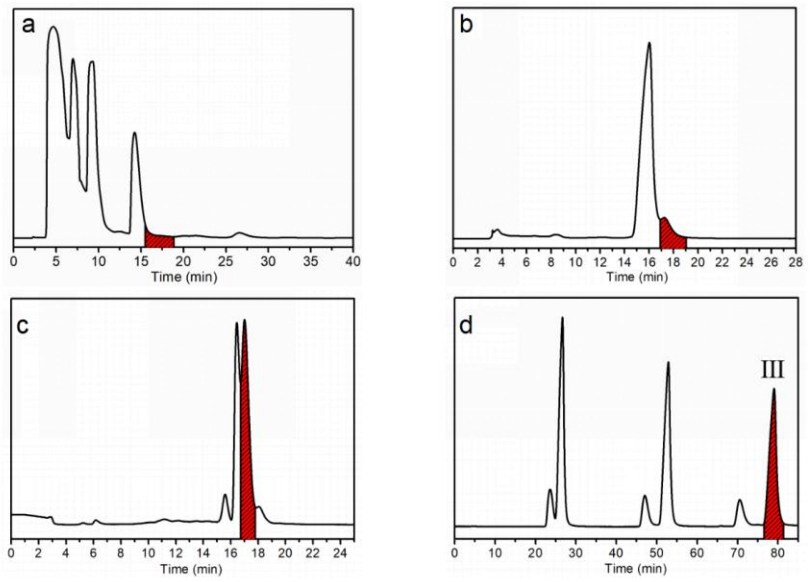


**Supplementary Figure 6. HPLC separation of *$C_{2v}$*-$C_{71}H_2$-III. a**) Column: buckyprep column (20 mm×250 mm), flow rate: 20 ml $min^{-1}$, eluent: toluene. **b**) Column: 5PBB column (10 mm×250 mm), flow rate: 6 ml $min^{-1}$, eluent: toluene. **c**) Column: buckyprep column (10 mm×250 mm), flow rate: 4 ml $min^{-1}$, eluent: toluene. **d**) Column: 5PBB column (10 mm×250 mm), flow rate: 4 ml $min^{-1}$, eluent: toluene. The regions of collected *$C_{2v}$*-$C_{71}H_2$-III containing components and pure *$C_{2v}$*-$C_{71}H_2$-III are highlighted as red shadow.

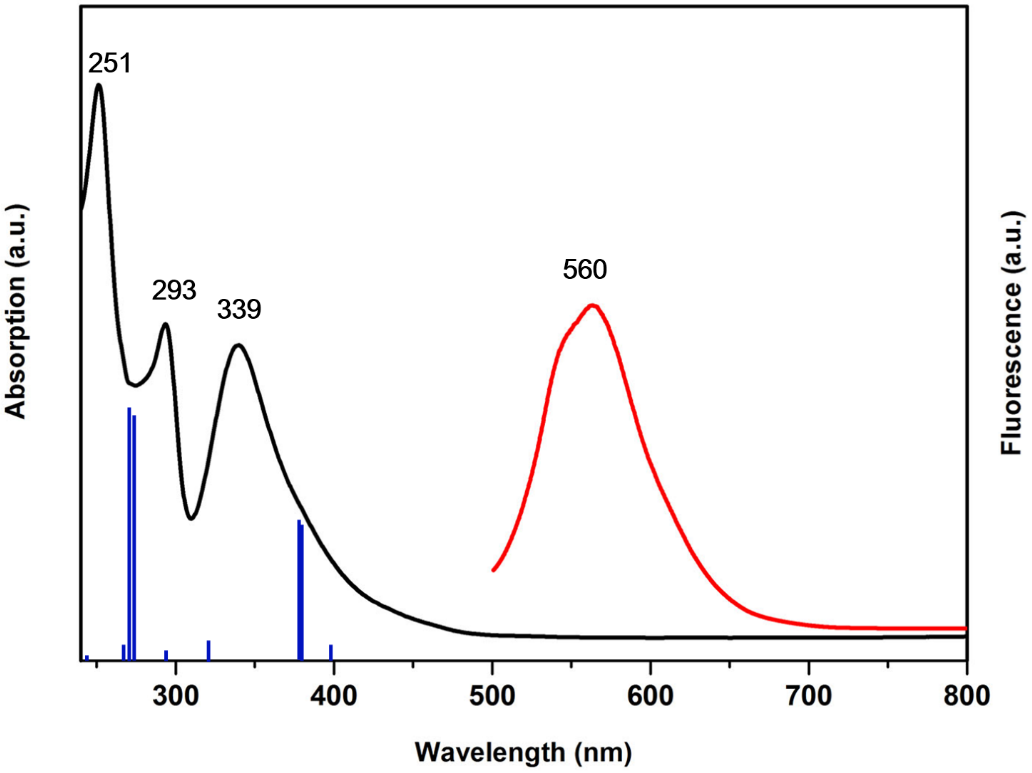


**Supplementary Figure 7.** UV-Vis absorption (black line) and normalized fluorescence emission (red line) spectrum of DPC (**2a**) in $CH_2Cl_2$ upon excitation at 465 nm, along with the oscillator strengths (blue bars) obtained by TD-DFT calculations.

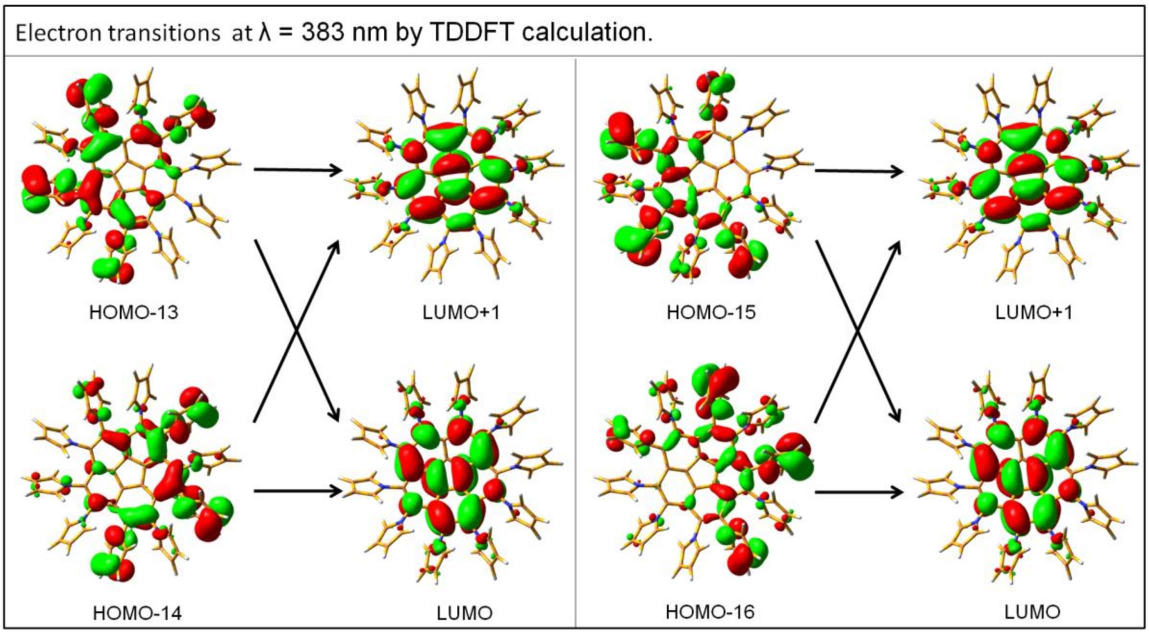


**Supplementary Figure 8.** A charge transfer transition band at λ = 383.79 nm and λ = 383.75 nm predicted by TD-DFT calculation.

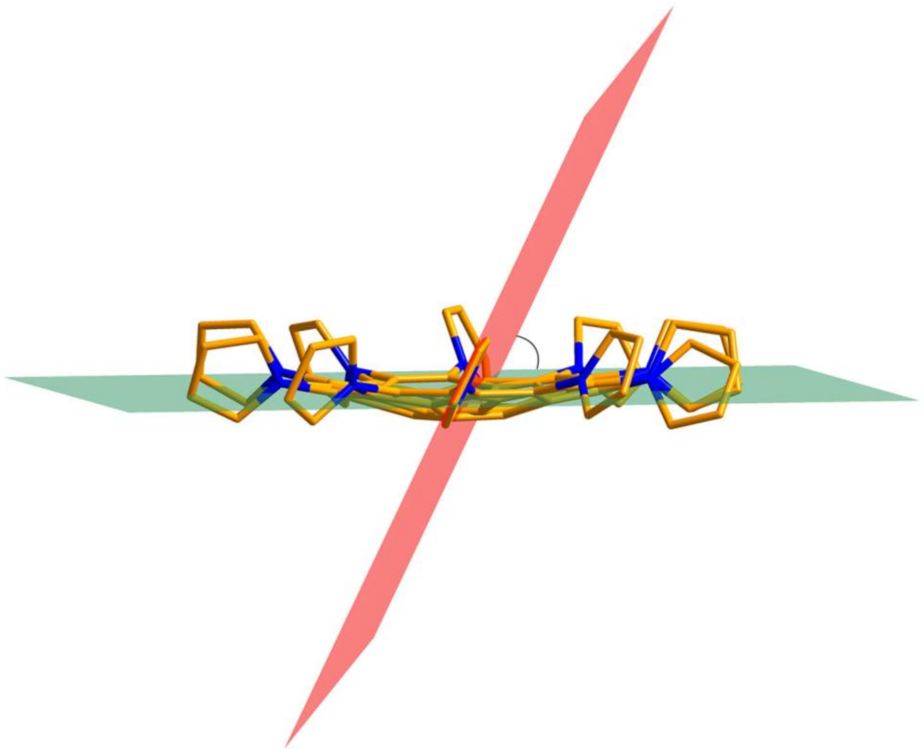

**Supplementary Figure 9.** Illustration dihedral angles between the plane of the pyrrole ring (represented in red) and the plane of five rims of corannulene (represented in green).

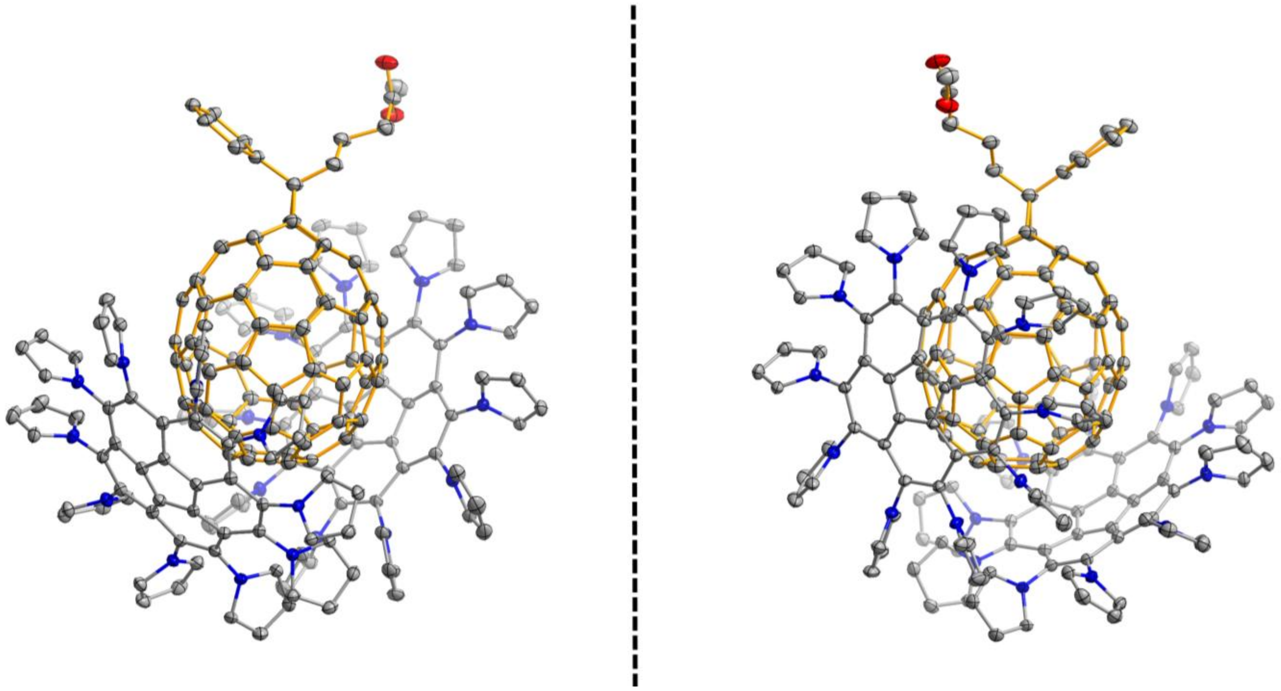

**Supplementary Figure 10.** A racemate of $\alpha$-$PC_{71}BM$ disordered with the occupancy of 0.5:0.5 between a pair of DPC hosts observed in the co-crystal of 2DPC{α-$PC_{71}BM$}.

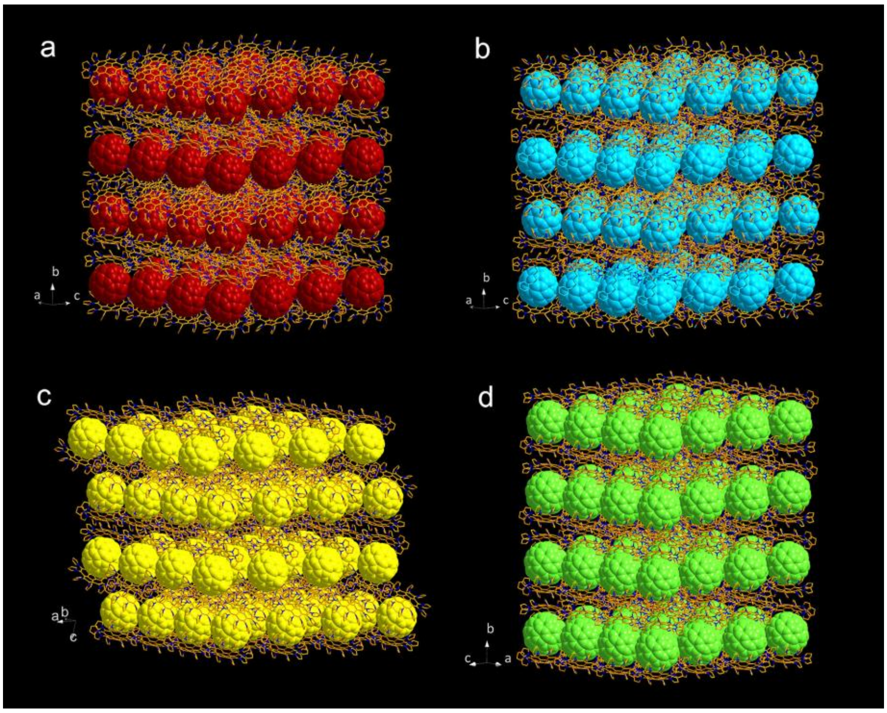


**Supplementary Figure 11.** Three dimensional structures of co-crystals between DPC **(2a)** and (a) $C_{70}$, (b) α-$PC_{71}BM$, (c) $C_{2v}$-$C_{71}H_2$-III and (d) $Sc_3N@C_{80}$.

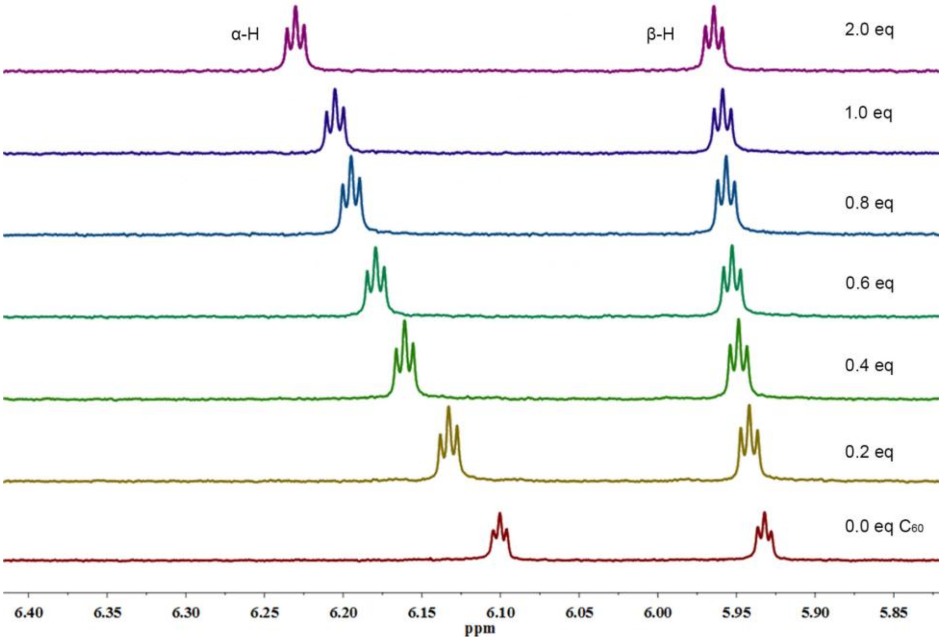


**Supplementary Figure 12.** $^1$H NMR chemical shift change for α, β hydrogen atoms of pyrryl groups in DPC (**2a**) vs variable concentrations of $C_{60}$.

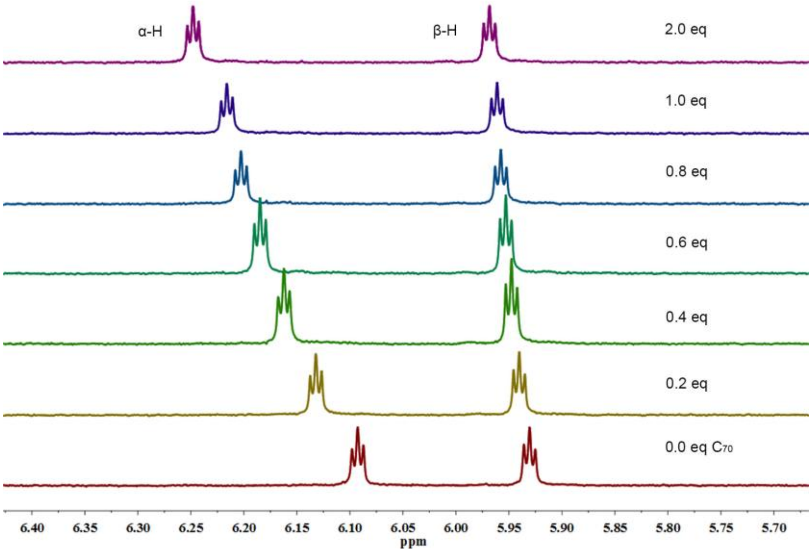


**Supplementary Figure 13.** $^1$H NMR chemical shift change for α, β hydrogen atoms of pyrryl groups in DPC (**2a**) vs variable concentrations of $C_{70}$.

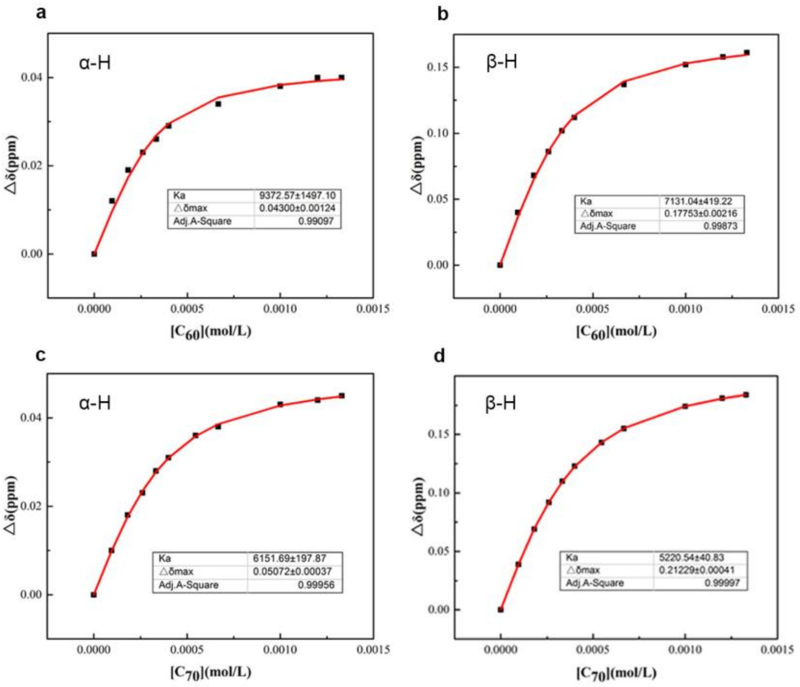


**Supplementary Figure 14. (a, b**) Changes of the chemical shifts for protons with the variable concentrations of $C_{60}$ in the supramolecular assembly between DPC (**2a**) and $C_{60}$ in deuterated toluene at 298 K. (**c, d**) Changes of the chemical shifts for protons with the variable concentrations of $C_{70}$ in the supramolecular assembly between DPC (**2a**) and $C_{70}$ in deuterated toluene at 298 K.

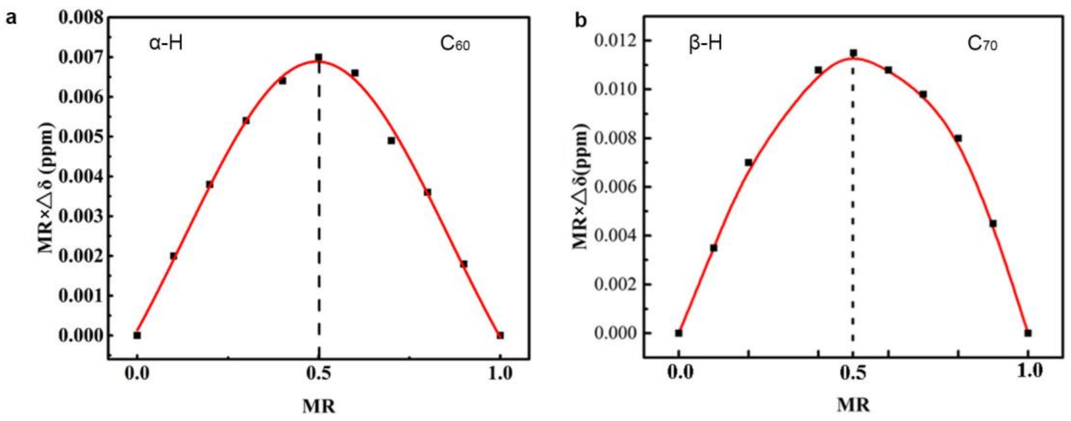


**Supplementary Figure 15.** (**a**) Job plot showing the 1:1 stoichiometry for DPC/$C_{60}$ supramolecule. MR = Molar ratio of **2a** and $C_{60}$. (**b**) Job plot showing the 1:1 stoichiometry for DPC/$C_{70}$ supramolecule. MR = Molar ratio of **2a** and $C_{70}$.

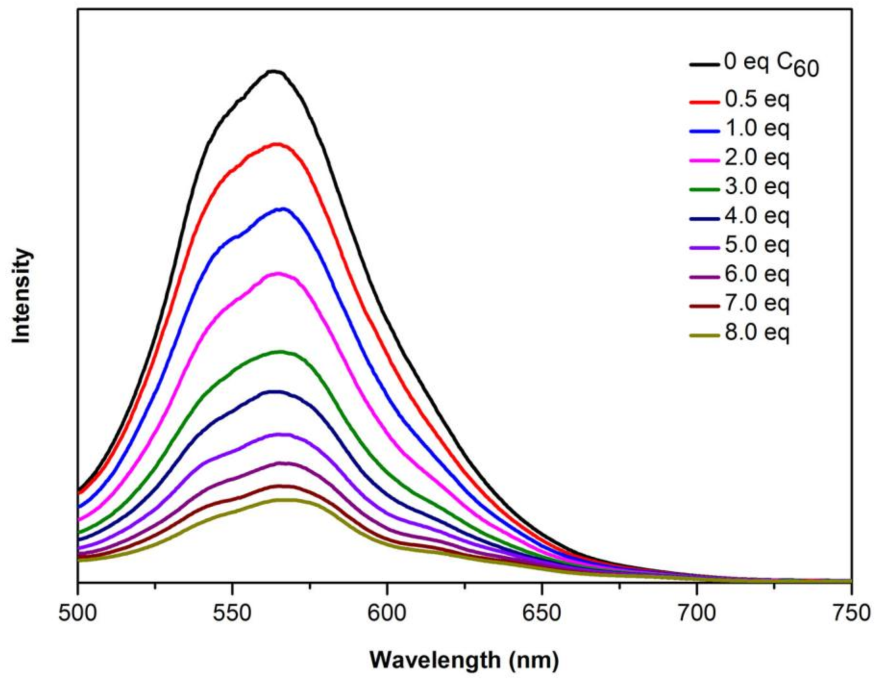


**Supplementary Figure 16.** Fluorescence spectra with gradual addition of 0.0-8.0 equiv. $C_{60}$ into a dichloromethane solution of **2a** ($2\times10^{-4}$ M).

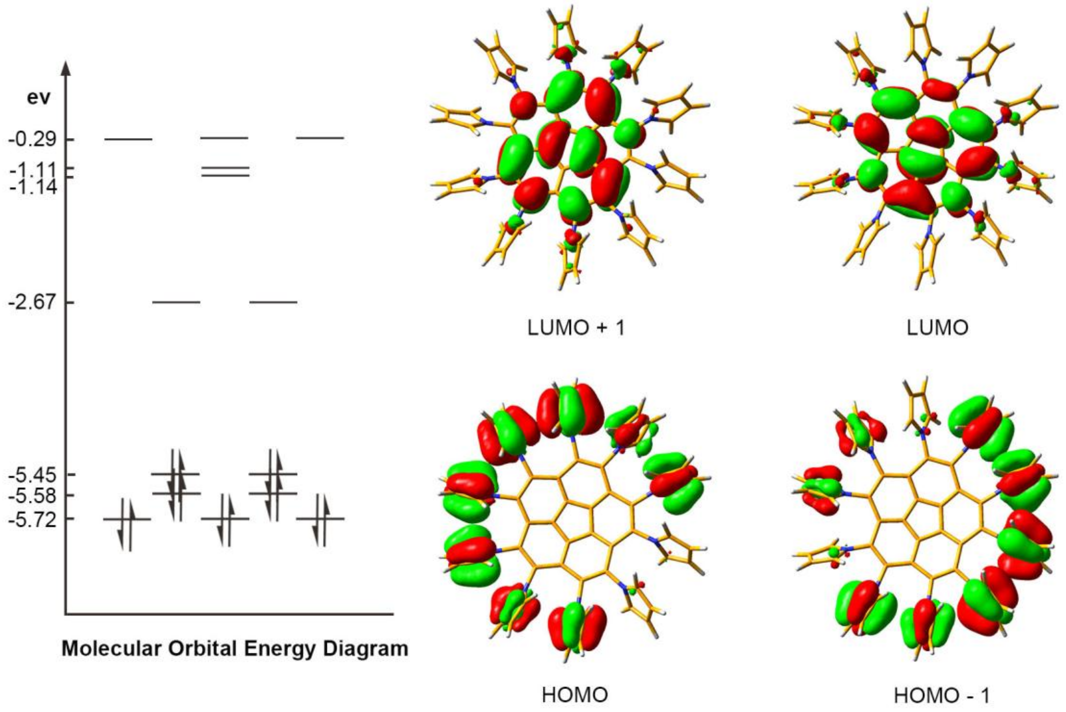


**Supplementary Figure 17.** Molecular orbitals (from HOMO-1 to LUMO+1) of **2a** calculated at B3LYP-D3BJ/6-31G(d, p) level.

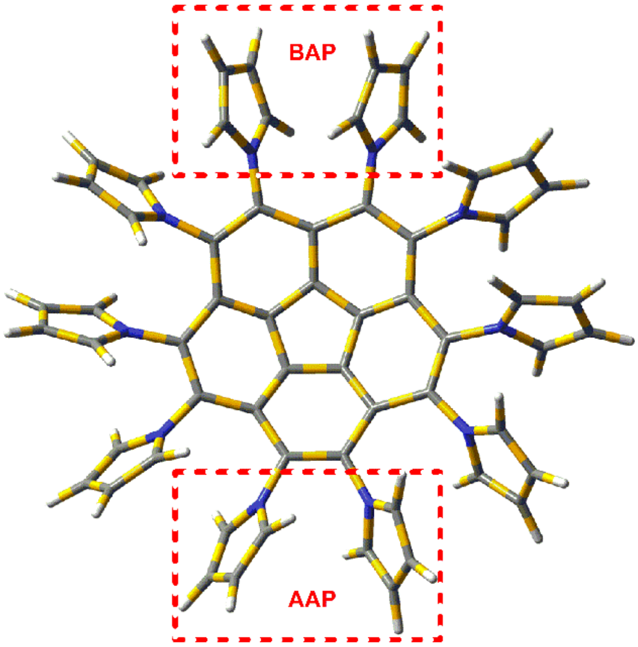


**Supplementary Figure 18.** Two types of adjacent pyrryl groups (AP) defined as AAP and BAP respectively.

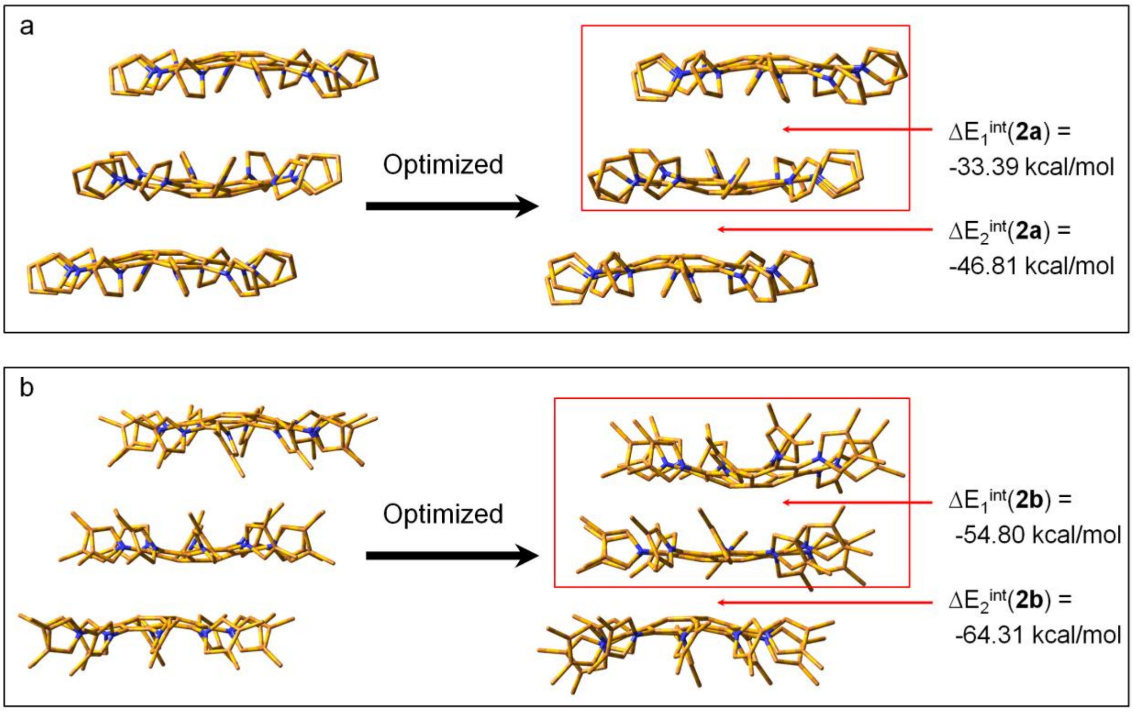


**Supplementary Figure 19.** Three molecular interaction systems of **2a** (or **2b**) are optimized by Gaussion 09 program at the B3LYP-D3BJ/6-31G(d) level. The interaction energies between each two molecules are presented.

## Supplementary Tables

**Supplementary Table 1.** UV-Vis absorption results of **2a** calculated by TD-DFT calculations at the B3LYP-D3BJ/6-31G(d) level of theory with dichloromethane solvent.

| E (eV) | λ (nm) | f | Composition of wave function |
|---|---|---|---|
| 3.1234 | 396.95 | 0.0595 | 0.11(H-12→L+1)+0.30(H-13→L)-0.15(H-13→L+1)-0.16(H-14→L)-0.33(H-14→L+1)+0.35(H-15→L)-0.31(H-16→L+1) |
| 3.1241 | 396.87 | 0.0585 | 0.12(H-12→L) +0.30(H-13→L+1)+0.15(H-13→L)+0.32(H-14→L)-0.17(H-14→L+1) +0.31(H-15→L+1)+0.35(H-16→L) |
| 3.2305 | 383.79 | 0.5152 | 0.34(H-13→L+1)+0.33(H-14→L)+0.17(H-15→L)-0.29(H-15→L+1)- 0.31(H-16→L)-0.19(H-16→L+1) |
| 3.2308 | 383.75 | 0.5241 | -0.32(H-13→L)+0.330.34(H-14→L+1)+ 0.29(H-15→L)+0.18(H-15→L+1) +0.170.31(H-16→L)-0.32(H-16→L+1) |
| 3.8669 | 320.63 | 0.0775 | 0.70(H-19→L) |
| 3.8708 | 320.31 | 0.0775 | 0.70(H-19→L+1) |
| 4.1958 | 295.50 | 0.0114 | 0.60(H-7→L+2)-0.14(H-7→L+3)-0.24(H-22→L) |
| 4.2226 | 293.62 | 0.0395 | 0.23(H-7→L+2)+0.55(H-7→L+3)-0.13(H-8→L+2)+0.24(H-10→L+3)+0.13 (H-22→L+1)+0.16(H-23→L) |
| 4.2307 | 293.06 | 0.0335 | 0.26(H-8→L+2)+0.52(H-8→L+3)+0.27(H-11→L+3)+0.12(H-22→L)-0.20(H-23→L+1) |
| 4.5812 | 270.64 | 0.9476 | 0.16(H-10→L+2)+0.11(H-10→L+3)+0.16(H-11→L+2)-0.33(H-11→L+3)+0.37(H-22→L)-0.37(H-23→L+1) |
| 4.5814 | 270.62 | 0.9156 | 0.15(H-10→L+2)-0.32(H-10→L+3)-0.16(H-11→L+2)-0.10(H-11→L+3)+0.37(H-22→L+1)+0.37(H-23→L) |
| 4.5846 | 270.44 | 0.0628 | 0.67(H-12→L+3) |
| 5.0724 | 244.43 | 0.0234 | 0.63(H-17→L+2)+0.15(H-10→L+4)+0.14(H-11→L+5) |
| 5.0785 | 244.13 | 0.0156 | 0.62(H-18→L+2)-0.15(H-10→L+5)+0.15(H-11→L+4) |

**Supplementary Table 2.** Detailed conditions for growth of co-crystals of DPC (**2a**) and fullerenes.

| **Crystals** | **Solvent for fullerenes and DPC** |
|---|---|
| 2DPC{$C_{60}$} | $C_{60}$ in $CS_2$ + DPC in $CH_2Cl_2$ |
| 2DPC{$C_{70}$} | $C_{60}$ in $C_7H_8$* + DPC in $C_7H_8$ or $CH_2Cl_2$ |
| 2DPC{$Sc_3N@C_{80}$} | $C_{60}$ in $CS_2$ + DPC in $CH_2Cl_2$ |
| 2DPC{$C_{90}$} | $C_{60}$ in $C_7H_8$ + DPC in $C_7H_8$ or $CH_2Cl_2$ |
| 2DPC{α-$PC_{71}BM$} | $C_{60}$ in $C_7H_8$ + DPC in $C_7H_8$ or $CH_2Cl_2$ |
| 2DPC{$\beta_1$-$PC_{71}BM$} | $C_{60}$ in $C_7H_8$ + DPC in $C_7H_8$ or $CH_2Cl_2$ |
| 2DPC{$PC_{61}P$} | $C_{60}$ in $C_7H_8$ + DPC in $C_7H_8$ or $CH_2Cl_2$ |
| 2DPC{$PC_{61}AE$} | $C_{60}$ in $C_7H_8$ + DPC in $C_7H_8$ or $CH_2Cl_2$ |
| 2DPC{$C_{60}HPh$} | $C_{60}$ in $C_7H_8$ + DPC in $C_7H_8$ or $CH_2Cl_2$ |
| 2DPC{$C_{60}HCH_3$} | $C_{60}$ in $C_7H_8$ + DPC in $C_7H_8$ or $CH_2Cl_2$ |
| 2DPC{$C_{71}H_2$-I} | $C_{60}$ in $C_7H_8$ + DPC in $C_7H_8$ or $CH_2Cl_2$ |
| 2DPC{$C_{71}H_2$-II} | $C_{60}$ in $C_7H_8$ + DPC in $C_7H_8$ or $CH_2Cl_2$ |
| 2DPC {$C_{71}H_2$-III} | $C_{60}$ in $C_7H_8$ + DPC in $C_7H_8$ or $CH_2Cl_2$ |
| 2DPC{$C_{65}H_6$} | $C_{60}$ in $C_7H_8$ + DPC in $C_7H_8$ or $CH_2Cl_2$ |
| 2DPC{$(C_{59}N)_2$} | $C_{60}$ in $C_7H_8$ + DPC in $C_7H_8$ or $CH_2Cl_2$ |

***** $C_7H_8$ represent the molecular formula of toluene.

**Supplementary Table 3.** All ten dihedral angles (°) of DPC **(2a)**.

| DPC (**2a**) | $\alpha_1$ | $\alpha_2$ | $\alpha_3$ | $\alpha_4$ | $\alpha_5$ | $\alpha_6$ | $\alpha_7$ | $\alpha_8$ | $\alpha_9$ | $\alpha_{10}$ |
|---|---|---|---|---|---|---|---|---|---|---|
| | 66.66 | 64.49 | 66.02 | 65.07 | 62.27 | 63.43 | 64.75 | 63.78 | 66.80 | 64.37 |

$\alpha_n$: Dihedral angles between the plane of the pyrryl ring and the plane of five rims of corannulene.

**Supplementary Table 4.** Crystallographic data collection and structure refinement statistics for DPC(**2a**), **2b**, 2DPC{$C_{60}$} and 2DPC{$C_{70}$}.

| | DPC (**2a**) | **2b** | 2DPC{$C_{60}$} | 2DPC{$C_{70}$} |
|---|---|---|---|---|
| empirical formula | $C_{60}H_{40}N_{10}$ | $C_{80}H_{80}N_{10}$ | $C_{181}H_{80}N_{20}S_2$ | $C_{190}H_{80}N_{20}$ |
| formula weight | 901.02 | 1181.54 | 2598.77 | 2642.74 |
| habit | block | block | block | block |
| *T*, K | 100 | 100 | 100 | 100 |
| crystal system | Monoclinic | Monoclinic | Monoclinic | Monoclinic |
| space group | $P\ 2_1/n$ | $P\ 2_1/c$ | $P\ 2_1/c$ | $P\ 2_1/c$ |
| *a*, Å | 11.2534(8) | 18.5320(11) | 14.2333(5) | 14.6364(4) |
| *b*, Å | 15.0384(12) | 24.2016(10) | 32.3849(12) | 32.6168(8) |
| *c*, Å | 27.4028(18) | 16.4860(9) | 28.2217(11) | 28.4617(8) |
| *α*, deg | 90 | 90 | 90 | 90 |
| *β*, deg | 96.211(6) | 110.734(6) | 94.184(4) | 94.657(2) |
| *γ*, deg | 90 | 90 | 90 | 90 |
| *V*, $Å^3$ | 4610.2(6) | 6915.2(7) | 12974.0(8) | 13542.5(6) |
| *Z* | 4 | 4 | 4 | 4 |
| *Dc*, $g/cm^3$ | 1.298 | 1.135 | 1.330 | 1.296 |
| *F*(000) | 1880.0 | 2520.0 | 5352.0 | 5440.0 |
| crystal size, $mm^3$ | 0.4×0.2×0.1 | 0.3×0.3×0.15 | 0.3×0.3×0.2 | 0.4×0.4×0.3 |
| $2\theta_{max}$, ° | 125.454 | 131.882 | 132.502 | 131.440 |
| $R_1$ ($I > 2\sigma(I)$) | 0.0939 | 0.0629 | 0.1074 | 0.0620 |
| $wR_2$ (all data) | 0.2564 | 0.1869 | 0.3499 | 0.1738 |
| Completeness (%) | 99.90 | 99.93 | 99.91 | 99.90 |
| Resolution (Å) | 0.87 | 0.84 | 0.84 | 0.85 |
| GOF | 1.108 | 1.031 | 1.040 | 1.034 |
| obs reflects | 5159 | 8652 | 11511 | 17801 |
| total reflects | 15149 | 26135 | 50307 | 50441 |
| parameters | 848 | 1184 | 2369 | 2703 |

**Supplementary Table 5.** Crystallographic data collection and structure refinement statistics for co-crystals of 2DPC{$C_{90}$}, 2DPC{α-$PC_{71}BM$}, 2DPC{$\beta_1$-$PC_{71}BM$}and 2DPC{$PC_{61}P$}.

| | 2DPC{$C_{90}$} | 2DPC{α-$PC_{71}BM$} | 2DPC{$\beta_1$-$PC_{71}BM$} | 2DPC{$PC_{61}P$}* |
|---|---|---|---|---|
| empirical formula | $C_{210}H_{80}N_{20}$ | $C_{209}H_{102}N_{20}O_2$ | $C_{202}H_{94}N_{20}O_2$ | $C_{194}H_{91}Cl_2N_{21}S_2$ |
| formula weight | 2882.94 | 2925.10 | 2832.97 | 2850.89 |
| habit | block | block | block | block |
| $T$, K | 100 | 100 | 100 | 100 |
| crystal system | Monoclinic | Monoclinic | Triclinic | Monoclinic |
| space group | $P\ 2_1$/n | $P\ 2_1$/c | $P$ -1 | $P\ 2_1$/c |
| $a$, Å | 14.9139(2) | 14.4916(7) | 20.7570(6) | 14.3185(6) |
| $b$, Å | 32.1663(6) | 32.7504(18) | 23.9255(6) | 32.2585(16) |
| $c$, Å | 33.2022(4) | 29.7049(17) | 31.8983(7) | 28.3686(11) |
| $\alpha$, deg | 90 | 90 | 69.564(2) | 90 |
| $\beta$, deg | 102.905(2) | 94.749(5) | 87.746(2) | 96.125(4) |
| $\gamma$, deg | 90 | 90 | 79.596(2) | 90 |
| $V$, Å$^3$ | 15525.6(4) | 14049.7(13) | 14595.5(7) | 13028.5(1) |
| $Z$ | 4 | 4 | 4 | 4 |
| $Dc$, g/cm$^3$ | 1.233 | 1.383 | 1.289 | 1.453 |
| $F$(000) | 5920.0 | 6048.0 | 5848.0 | 5872.0 |
| crystal size, mm$^3$ | 0.2×0.15×0.1 | 0.3×0.3×0.2 | 0.2×0.15×0.12 | 0.15×0.12×0.1 |
| $2\theta_{max}$, ° | 125.474 | 132.754 | 132.514 | 131.416 |
| $R_1$ ($I > 2\sigma(I)$) | 0.1270 | 0.0777 | 0.0960 | 0.0918 |
| $wR_2$ (all data) | 0.3268 | 0.2483 | 0.2879 | 0.2750 |
| Completeness (%) | 99.96 | 99.83 | 99.92 | 89.23 |
| Resolution (Å) | 0.87 | 0.84 | 0.84 | 0.85 |
| GOF | 1.051 | 1.036 | 1.015 | 1.025 |
| obs reflects | 16708 | 15409 | 32930 | 14212 |
| total reflects | 24372 | 54437 | 49382 | 19506 |
| parameters | 2071 | 2780 | 4822 | 1981 |

***** Explanation for an alert level A: some diffraction spots were not obtained likely due to overexposure at the low angle diffraction during the test.

**Supplementary Table 6.** Crystallographic data collection and structure refinement statistics for 2DPC{$PC_{61}AE$}, 2DPC{$C_{71}H_2$-I}, 2DPC{$C_{71}H_2$-II} and 2DPC{$C_{65}H_6$}.

| | 2DPC{$PC_{61}AE$} | 2DPC{$C_{71}H_2$-I} | 2DPC{$C_{71}H_2$-II} | 2DPC{$C_{65}H_6$}[a] |
|---|---|---|---|---|
| empirical formula | $C_{190}H_{91}N_{21}O_2$ | $C_{212}H_{106}N_{20}$ | $C_{219}H_{114}N_{20}$ | $C_{206}H_{110}N_{20}$ |
| formula weight | 2699.83 | 2933.16 | 3025.30 | 2865.13 |
| habit | block | block | block | block |
| *T*, K | 100 | 100 | 100 | 273 |
| crystal system | Monoclinic | Triclinic | Triclinic | Triclinic |
| space group | $P\ 2_1/c$ | *P* -1 | *P* -1 | *P* -1 |
| *a*, Å | 14.2672(2) | 14.8351(6) | 14.4222(5) | 18.9031(6) |
| *b*, Å | 32.1131(6) | 17.4182(8) | 17.4610(5) | 19.3035(5) |
| *c*, Å | 28.4039(6) | 30.0997(7) | 18.1172(7) | 21.0492(7) |
| $\alpha$, deg | 90 | 78.223(3) | 113.558(3) | 82.445(1) |
| $\beta$, deg | 94.071(2) | 78.217(3) | 106.695(3) | 74.754(1) |
| $\gamma$, deg | 90 | 69.831(4) | 98.916(2) | 65.150(1) |
| *V*, $Å^3$ | 12980.8(4) | 7071.0(5) | 3812.0(2) | 6722.4(4) |
| *Z* | 4 | 2 | 1 | 2 |
| *Dc*, $g/cm^3$ | 1.381 | 1.378 | 1.318 | 1.415 |
| *F*(000) | 5576.0 | 3036.0 | 1568.0 | 2972.0 |
| crystal size, $mm^3$ | 0.15×0.12×0.1 | 0.2×0.15×0.1 | 0.2×0.15×0.1 | 0.2×0.15×0.12 |
| $2\theta_{max}$, ° | 131.552 | 125.6 | 125.588 | 50.726 |
| $R_1$ ($I > 2\sigma(I)$) | 0.0870 | 0.0815 | 0.0547 | 0.0738 |
| $wR_2$ (all data) | 0.2564 | 0.2383 | 0.1485 | 0.2005 |
| Completeness (%) | 99.83 | 99.74 | 99.85 | 94.20 |
| Resolution (Å) | 0.85 | 0.84 | 0.85 | 0.83 |
| GOF | 1.025 | 1.015 | 1.037 | 1.035 |
| obs reflects | 16863 | 15886 | 10382 | 20116 |
| total reflects | 21900 | 22587 | 12205 | 23230 |
| parameters | 2578 | 2273 | 1398 | 3587 |

[a] X-ray single-crystal diffraction data of 2DPC{$C_{65}H_6$} were collected with a Mo Kα (λ = 0.71073 Å) microfocus X-ray source.

**Supplementary Table 7.** Crystallographic data collection and structure refinement statistics for 2DPC{$C_{60}$HPh}, 2DPC{$C_{60}HCH_3$}, 2DPC{$C_{71}H_2$-III}, 2DPC{$Sc_3NC_{80}$}and 2DPC{$(C_{59}N)_2$.

| | 2DPC{$C_{60}$HPh} | 2DPC{$C_{60}HCH_3$}* | 2DPC{$C_{71}H_2$-III} | 2DPC{$Sc_3NC_{80}$} | 2DPC{$(C_{59}N)_2$ |
|---|---|---|---|---|---|
| empirical formula | $C_{186}H_{86}N_{20}$ | $C_{181}H_{84}N_{20}$ | $C_{205}H_{98}N_{20}$ | $C_{203}H_{86}Cl_6N_{21}Sc_3$ | $C_{238}H_{80}N_{22}$ |
| formula weight | 2600.74 | 2538.68 | 2841.03 | 3166.50 | 3247.24 |
| habit | block | block | block | block | block |
| *T*, K | 100 | 100 | 150 | 100 | 293 |
| crystal system | Monoclinic | Monoclinic | triclinic | triclinic | orthorhombic |
| space group | $P\ 2_1/c$ | $P\ 2_1/c$ | *P* -1 | *P* -1 | *P* na21 |
| *a*, Å | 14.2514(3) | 14.2282(1) | 14.7974(4) | 14.5217(4) | 21.1466(2) |
| *b*, Å | 32.2390(7) | 32.5590(3) | 17.0642(4) | 18.2551(5) | 28.6170(3) |
| *c*, Å | 28.2387(7) | 28.1984(2) | 30.7179(7) | 28.9155(7) | 28.3370(4) |
| *α*, deg | 90 | 90 | 78.188(2) | 101.095(2) | 90 |
| *β*, deg | 94.743(2) | 93.494(1) | 78.421(2) | 92.007(2) | 90 |
| *γ*, deg | 90 | 90 | 71.184(2) | 101.189(2) | 90 |
| *V*, $Å^3$ | 12929.9(5) | 13038.8(2) | 7109.4(3) | 7358.2(3) | 17148.2(3) |
| *Z* | 4 | 4 | 2 | 2 | 4 |
| *Dc*, g/$cm^3$ | 1.336 | 1.293 | 1.327 | 1.429 | 1.258 |
| *F*(000) | 5368.0 | 5240.0 | 2936 | 3232.0 | 6648.0 |
| crystal size, $mm^3$ | 0.2×0.15×0.1 | 0.15×0.12×0.1 | 0.3×0.2×0.2 | 0.5×0.5×0.5 | 0.7×0.5×0.5 |
| $2\theta_{max}$, ° | 127.38 | 126.876 | 131.628 | 131.298 | 124.994 |
| $R_1$ ($I>2\sigma(I)$) | 0.0794 | 0.0747 | 0.1147 | 0.1161 | 0.1237 |
| $wR_2$ (all data) | 0.2392 | 0.2100 | 0.3166 | 0.3187 | 0.2994 |
| Completeness (%) | 99.88 | 99.96 | 99.96 | 99.64 | 99.66 |
| Resolution (Å) | 0.86 | 0.86 | 0.85 | 0.85 | 0.86 |
| GOF | 1.025 | 1.025 | 1.039 | 1.018 | 1.195 |
| obs reflects | 14540 | 17121 | 18965 | 19826 | 16139 |
| total reflects | 21185 | 21011 | 112183 | 47826 | 20379 |
| parameters | 2450 | 2911 | 2274 | 2386 | 2342 |

**Supplementary Table 8.** The occupancies of DPC (**2a**), **2b** and 2DPC{fullerenes}

| Crystals | Fullerene Cage | DPC hosts |
|---|---|---|
| DPC (**2a**) | **-** | 0.573/0.427 |
| **2b** | - | 0.517/0.483 |
| 2DPC{$C_{60}$} | 0.514/0.486 | Ordered |
| 2DPC{$C_{70}$} | 0.847/0.153 | one of them is disordered with the occupancy of 0.733/0.267 |
| 2DPC{$C_1$(30)-$C_{90}$} | The occupancy of the cage is refined to be 100% with some SADI and DFIX commands | Ordered |
| 2DPC{$C_{71}H_2$-I} | ordered | one of them is disordered with the occupancy of 0.773/0.227 |
| 2DPC{$C_{71}H_2$-II} | The occupancy of the cage is refined to be 100% with some SADI and DFIX commands | Ordered |
| 2DPC{$C_{71}H_2$-III} | The occupancy of the cage is refined to be 100% with some SADI and DFIX commands | one of them is disordered with the occupancy of 0.603/0.397 |
| 2DPC{$C_{61}H_4$} | 0.5/0.25/0.25 | Ordered |
| 2DPC{$C_{65}H_6$} | 0.45/0.25/0.3 | one of them is disordered with the occupancy of 0.620/0.380 |
| 2DPC{$C_{66}H_6$} | 0.640/0.360 | Ordered |
| 2DPC{α-$PC_{71}BM$} | 0.518/0.482 | Ordered |
| 2DPC{β-$PC_{71}BM$} | Two fullerene sites are presented in the crystal, one of them is disordered with the occupancy of 0.521/0.479 | Ordered |
| 2DPC{$PC_{61}AE$} | 0.676/0.324 | Ordered |
| 2DPC{$PC_{61}P$} | Ordered | Ordered |
| 2DPC{$(C_{59}N)_2$} | The occupancy of the cage is refined to be 100% with some DFIX commands | Ordered |
| 2DPC{$Sc_3NC_{80}$} | The occupancy of the cage is refined to be 100% with some DFIX commands. For the $Sc_3N$ unit, the nitrogen atom is ordered, but the scandium atoms show some sort of disorder with the occupancy of 0.4/0.2/0.2/0.1/0.1 | one of them is disordered with the occupancy of 0.705/0.295 |

**Supplementary Table 9.** Twenty dihedral angles (°) of the chiral (+) host and (-) host in representative co-crystals of 2DPC{fullerene}.

| | Dihedral angle (°) | | | | | | | | | |
|---|---|---|---|---|---|---|---|---|---|---|
| 2DPC{$C_{60}$} | 63.382 | 66.928 | 66.490 | 64.544 | 64.437 | 63.995 | 64.342 | 62.858 | 64.919 | 59.623 |
| | 66.669 | 65.862 | 63.654 | 68.609 | 57.074 | 60.196 | 67.276 | 65.221 | 61.538 | 62.645 |
| 2DPC{$C_{70}$} | 63.125 | 61.012 | 61.871 | 58.335 | 64.103 | 67.054 | 64.845 | 64.270 | 66.986 | 68.287 |
| | 59.039 | 61.836 | 69.631 | 65.908 | 59.341 | 61.181 | 64.552 | 64.660 | 64.136 | 65.751 |
| 2DPC{α-$PC_{71}BM$} | 61.986 | 63.651 | 61.875 | 64.881 | 61.935 | 62.032 | 66.225 | 63.511 | 62.383 | 62.386 |
| | 67.517 | 65.954 | 60.686 | 63.004 | 62.598 | 61.407 | 65.145 | 68.108 | 58.046 | 62.870 |
| 2DPC{$Sc_3N@C_{80}$} | 62.093 | 63.991 | 63.956 | 65.833 | 67.513 | 67.518 | 64.351 | 59.343 | 62.167 | 65.058 |
| | 65.381 | 66.674 | 63.344 | 60.935 | 68.578 | 64.960 | 64.908 | 65.154 | 60.194 | 60.464 |
| 2DPC{$C_{2v}$-$C_{71}H_2$} | 66.425 | 61.786 | 61.091 | 65.801 | 66.878 | 66.307 | 62.559 | 62.657 | 63.270 | 62.835 |
| | 58.848 | 60.549 | 67.017 | 67.155 | 61.579 | 64.587 | 61.225 | 61.626 | 64.445 | 68.100 |
| 2DPC{$(C_{59}N)_2$} | 65.985 | 68.010 | 61.607 | 62.085 | 68.697 | 65.632 | 59.669 | 60.922 | 64.166 | 65.228 |
| | 64.194 | 66.085 | 66.538 | 60.290 | 61.054 | 65.588 | 68.720 | 70.429 | 64.521 | 65.618 |

**Supplementary Table 10.** The selected crystal data of representative co-crystals between DPC host (**2a**) and different types of fullerenes.

| Co-crystal with **2a** | α[a] | $d_l$[b] | $d_r$[c] | $D_l$[d] | $D_r$[e] | $L_l$[f] | $L_r$[g] |
|---|---|---|---|---|---|---|---|
| $C_{60}$ | 58.02 ° | 0.573Å | 0.570Å | 6.693Å | 6.637 Å | 3.258Å | 3.233Å |
| $C_{70}$ | 56.31 ° | 0.567Å | 0.578Å | 6.874Å | 6.941Å | 3.127Å | 3.240Å |
| $C_{90}$ | 68.55 ° | 0.568Å | 0.564Å | 7.708Å | 7.650Å | 3.399Å | 3.367Å |
| α-$PC_{71}BM$ | 58.49 ° | 0.545Å | 0.581Å | 6.867 Å | 7.072Å | 3.330Å | 3.132Å |
| $C_{2v}$-$C_{71}H_2$ | 57.80 ° | 0.579Å | 0.584Å | 6.965Å | 7.201Å | 3.375Å | 3.345Å |
| $Sc_3N@C_{80}$ | 1.49 ° | 0.567Å | 0.572Å | 7.340Å | 7.347Å | 3.374Å | 3.334Å |
| $(C_{59}N)_2$ | 0.44 ° | 0.577Å | 0.606Å | 6.710Å | 6.614Å | 3.274Å | 3.291Å |

[a] The dihedral angle between the plane of five rims of (+)DPC and (-)DPC.

[b] Bowl depth of (+)DPC which is defined as the distance between the plane of five corannulene rims and the plane of five membered ring of corannulene.

[c] Bowl depth of (-)DPC which is defined as the distance between the plane of five corannulene rims and the plane of five membered ring of corannulene .

[d] The distance between the centroid of buckyball and the five membered ring of (+)DPC.

[e] The distance between the centroid of buckyball and the five membered ring of (-)DPC.

[f] The nearest distance between the buckylball surface and the concave surface of (+)DPC.

[g] The nearest distance between the buckylball surface and the concave surface of (-)DPC.

**Supplementary Table 11.** Intramolecular interaction energy of **DPC** (**2a**) and its geometric configuration parameters.

| | AAP1 | AAP2 | AAP3 | AAP4 | AAP5 |
|---|---|---|---|---|---|
| d(Å) [a] | 2.745 | 2.742 | 2.735 | 2.733 | 2.737 |
| β(°) [b] | 41.49 | 41.62 | 41.59 | 41.58 | 41.67 |
| $\Delta E^{int}_{AAP}$(kcal/mol) [c] | 3.53 | 3.52 | 3.52 | 3.52 | 3.52 |
| | BAP1 | BAP2 | BAP3 | BAP4 | BAP5 |
| d(Å) [a] | 3.033 | 3.048 | 3.052 | 3.049 | 3.033 |
| β(°) [b] | 22.33 | 22.40 | 22.33 | 22.44 | 22.30 |
| $\Delta E^{int}_{BAP}$(kcal/mol) [c] | 1.56 | 1.59 | 1.56 | 1.55 | 1.59 |

[a] d represents the distance between two nitrogen atoms of adjacent pyrryl groups;

[b] β stands for the dihedral angle of the two adjacent pyrryl group planes;

[c] $\Delta E^{int}_{AAP}$ and $\Delta E^{int}_{BAP}$ is defined as the intramolecular interaction energy between AAP or BAP.

**Supplementary Table 12.** The results of ab initio calculations for bowl-to-bowl inversion barrier.

| | | Corannulene | Sumanene | DPC (+) | DPC (-) |
|---|---|---|---|---|---|
| SCF energy ( hartrees ) | GS | -768.247918014 | -807.539084653 | -2858.16514422 | -2858.16512995 |
| | TS | -768.231244529 | -807.508337397 | -2858.16019211 | -2858.16029990 |
| Inversion Barrier ( kcal/mol ) | | 10.46 | 19.29 | 3.11 | 3.03 |

Here GS represents the ground state of target bowl-shaped molecule and TS stands for the transition state of a planar conformation. The inversion barrier energy is defined as the self-consistent field (SCF) energy difference between GS and TS[7].

**Supplementary Table 13.** The interaction energies (kcal/mol) of representative six co-crystals in Fig. 6.

| | $C_{60}$ | $C_{70}$ | $Sc_3N@C_{80}$ | $(C_{59}N)_2$ | $C_{2v}$-$C_{71}H_2$ | α-$PC_{71}BM$ |
|---|---|---|---|---|---|---|
| $\Delta E^{int}_{F/2DPC}$ [a] | -62.60 | -66.01 | -70.13 | -76.54 | -65.71 | -71.09 |
| $\Delta E^{int}_{F/(+)DPC}$ | -31.44 | -32.70 | -35.30 | -38.55 | -33.75 | -33.16 |
| $\Delta E^{int}_{F/(-)DPC}$ | -31.39 | -33.57 | -35.07 | -37.94 | -32.31 | -38.18 |
| $\Delta E^{int}_{(+)DPC/(-)DPC}$ [b] | -10.53 | -10.49 | -0.26 | -0.23 | -9.48 | -10.25 |

[a] $\Delta E^{int}_{F/2DPC}$ stands for the interaction energy between fullerene (F) and both (+)DPC and (-)DPC.

[b] $\Delta E^{int}_{(+)DPC/(-)DPC}$ represents the interaction energy between (+)DPC and (-)DPC.

**Supplementary Table 14.** The interaction energies (kcal/mol) between fullerene and pyrryl groups/corannulene core using fragmentation methods by capping the dangling bonds with H atoms.

| | $C_{60}$ | $C_{70}$ | $Sc_3N@C_{80}$ | $(C_{59}N)_2$ | $C_{2v}$-$C_{71}H_2$ | α-$PC_{71}BM$ |
|---|---|---|---|---|---|---|
| $\Delta E^{int}_{F/2DPC}$ | -62.60 | -66.01 | -70.13 | -76.54 | -65.71 | -71.09 |
| $\Delta E^{int}_{F/P}$ | -44.85 | -47.74 | -54.93 | -59.97 | -48.12 | -53.57 |
| $\Delta E^{int}_{F/C}$ | -28.97 | -29.87 | -29.72 | -30.38 | -29.02 | -29.83 |
| $\Delta E$corr | 11.22 | 11.60 | 14.52 | 13.81 | 11.43 | 12.31 |

Here $\Delta E^{int}_{F/2DPC}$, $\Delta E^{int}_{F/P}$, $\Delta E^{int}_{F/C}$ stand for the total interaction energy between fullerene (F) and both hosts (2DPC), fullerene (F) and pyrryl groups (P), and fullerene (F) and corannulene part (C) respectively, and $\Delta E^{corr}$ is defined as two-body interaction correlation energy between pyrryl groups and corannulene core.

# Supplementary References


1. Seiders T. J., Baldridge K. K., Elliott E. L., Grube G. H., Siegel J. S. Synthesis and quantum mechanical structure of sym-pentamethylcorannulene and decamethylcorannulene. *J. Am. Chem. Soc.* **121,** 7439-7440 (1999).
2. Thordarson, P. Determining association constants from titration experiments in supramolecular chemistry. *Chem. Soc. Rev.* **40**, 1305-1323 (2011).
3. Boys, S. F., Bernardi, F. The calculation of small molecular interactions by the differences of separate total energies. Somprocedures with reduced errors. *Mol. Phys.* **100**, 65-73 (2002).
4. Grimme, S., Ehrlich, S., Goerigk, L. Effect of the damping function in dispersion corrected density functional theory. *J. Comput. Chem.* **32**, 1456-1465 (2011).
5. Frisch, M. J., Trucks, G. W., Schlegel, H. B., Scuseria, G. E., Robb, M. A., Cheeseman, J. R., Scalmani, G., Barone, V., Mennucci, B., Petersson, G. A., Nakatsuji, H., Caricato, M., Li, X., Hratchian, H. P., Izmaylov, A. F., Bloino, J., Zheng, G., Sonnenberg, J. L., Hada, M., Ehara, M., Toyota, K., Fukuda, R., Hasegawa, J., Ishida, M., Nakajima, T., Honda, Y., Kitao, O., Nakai, H., Vreven, T., Montgomery, Jr., J. A., Peralta, J. E., Ogliaro, F., Bearpark, M., Heyd, J. J., Brothers, E., Kudin, K. N., Staroverov, V. N., Keith, T., Kobayashi, R., Normand, J., Raghavachari, K., Rendell, A., Burant, J. C., Iyengar, S. S., Tomasi, J., Cossi, M., Rega, N., Millam, J. M., Klene, M., Knox, J. E., Cross, J. B., Bakken, V., Adamo, C., Jaramillo, J., Gomperts, R., Stratmann, R. E., Yazyev, O., Austin, A. J., Cammi, R., Pomelli, C., Ochterski, J. W., Martin, R. L., Morokuma, K., Zakrzewski, V. G., Voth, G. A., Salvador, P., Dannenberg, J. J., Dapprich, S., Daniels, A. D., Farkas, O., Foresman, J. B., Ortiz, J. V., Cioslowski, J. & Fox, D. J. Gaussian 09, Revision D.01. (2016).
6. Su, P., Chen, Z., Wu, W. An energy decomposition analysis study for intramolecular non-covalent interaction. *Chem. Phys. Lett.* **635**, 250-256 (2015).
7. Sygula, A., Rabideau, P. W. Bowl-to-bowl inversion in polynuclear aromatic hydrocarbons with curved surfaces: an ab initio study. *J. Chem. Soc. Chem. Commun.* **12**, 1497-1499 (1994).
