# Supplementary material for: Flexible decapyrrylcorannulene hosts
Source: Nat Commun. 2019 Jan 30;10:485. doi: 10.1038/s41467-019-08343-6 (PMC6353959; doi:10.1038/s41467-019-08343-6)
Supplement: Supplementary file 3 — Description of Additional Supplementary Files [file 41467_2019_8343_MOESM3_ESM.pdf]

File name:

Supplementary Data 1

Description:

Cartesian coordinates of the calculated molecules at the B3LYP-D3BJ/6-31G(d)) level.
